# The Emergence of Tuning to Global Shape Properties of Radial Frequency Patterns in the Ventral Visual Pathway

**DOI:** 10.1523/JNEUROSCI.2237-22.2023

**Published:** 2023-07-19

**Authors:** Samuel J. D. Lawrence, Elisa Zamboni, Richard J. W. Vernon, André D. Gouws, Alex R. Wade, Antony B. Morland

**Affiliations:** ^1^Department of Psychology, University of York, York, UK, YO10 5DD; ^2^York Neuroimaging Centre, University of York, York, UK, YO10 5NY; ^3^ York Biomedical Research Institute, University of York, York, UK, YO10 5DD

**Keywords:** global processing, lateral occipital cortex, radial frequency, shape processing, V4, visual cortex

## Abstract

Radial frequency (RF) patterns, created by sinusoidal modulations of a circle's radius, are processed globally when RF is low. These closed shapes therefore offer a useful way to interrogate the human visual system for global processing of curvature. RF patterns elicit greater responses than those to radial gratings in V4 and more anterior face-selective regions of the ventral visual pathway. This is largely consistent with work on nonhuman primates showing curvature processing emerges in V4, but is evident also higher up the ventral visual stream. Rather than contrasting RF patterns with other stimuli, we presented them at varied frequencies in a regimen that allowed tunings to RF to be derived from 8 human participants (3 female). We found tuning to low RF in lateral occipital areas and to some extent in V4. In a control experiment, we added a high-frequency ripple to the stimuli disrupting the local contour. Low-frequency tuning to these stimuli remained in the ventral visual stream, underscoring its role in global processing of shape curvature. We then used representational similarity analysis to show that, in lateral occipital areas, the neural representation was related to stimulus similarity, when it was computed with a model that captured how stimuli are perceived. We therefore show that global processing of shape curvature emerges in the ventral visual stream as early as V4, but is found more strongly in lateral occipital regions, which exhibit responses and representations that relate well to perception.

**SIGNIFICANCE STATEMENT** We show that tuning to low radial frequencies, known to engage global shape processing mechanisms, was localized to lateral occipital regions. When low-level stimulus properties were accounted for such tuning emerged in V4 and LO2 in addition to the object-selective region LO. We also documented representations of global shape properties in lateral occipital regions, and these representations were predicted well by a proxy of the perceptual difference between the stimuli.

## Introduction

Processing of increasingly complex spatial features occurs up the hierarchy of the ventral visual pathway. At the start of the pathway, V1 processes orientation (Hubel and Wiesel, 1968), while areas high up the pathway respond selectively to real-world objects (Tanaka, 1996; Grill-Spector et al., 2001) or faces (Desimone et al., 1984; Kanwisher et al., 1996; Tsao et al., 2006). The intermediate levels of the pathway process spatial features, such as curvature (Kourtzi and Connor, 2011), which has long been seen as part of pattern vision (Riggs, 1973) and is the focus of the present paper.

Pioneering work assessed responses in the intermediate area V4, where many cells had response preferences to polar and hyperbolic stimuli, which exhibited curvature, over the straight lines of cartesian stimuli (Gallant et al., 1993, 1996). Others have shown in experiments using angles and curves (Pasupathy and Connor, 1999) and simple curved shapes (Pasupathy and Connor, 2001) how V4 neurons are selective to boundary conformations (Pasupathy et al., 2020). V4 neurons also respond to other information (Zeki, 1973) with domains specific to curvature, orientation, and color being evident (Conway et al., 2007; Roe et al., 2012; Hu et al., 2020; Tang et al., 2020). Experiments using naturalistic visual stimuli, from which curvature was quantified, have shown that curvature processing is not limited to V4 and that two regions more anterior in the ventral visual pathway also respond to curvature in macaque (Yue et al., 2014). V4 has therefore been referred to as “curvature emergent” as areas antecedent to it do not appear to process curvature (Hu et al., 2020).

In humans, a role for V4 and other extrastriate areas in processing curvature has also been found. Wilkinson et al., found greater responses in V4 to concentric than parallel patterns, suggesting that there was a preference for curvature in V4 (Wilson et al., 1997). However, it was only in the more anterior fusiform face area (FFA) that responses to the concentric, curved patterns were greater than the straight lined radial patterns. A preference for concentric curvature (defined in arrays of Gabors) was found in V4, but also earlier in V3 (Dumoulin and Hess, 2007). We have found that shape curvature representations emerge in areas LO1 and LO2, which could be considered as one step further up the object processing pathway than V4 (Vernon et al., 2016). Yue et al. (2020) found preferences to curvature in V4 and also to some extent in V3 and also in regions that were more anterior in the ventral visual pathway, where more complex aspects of curvature were processed (Yue et al., 2014). The lateral occipital complex (LOC) (Malach et al., 1995; Grill-Spector et al., 2001) has also been found to have a shape representation based on shape features (Haushofer et al., 2008; Op de Beeck et al., 2008; Drucker and Aguirre, 2009). There is, therefore, reasonably broad agreement between there being an emergence of curvature processing in the human and macaque brain in V4 and that curvature information is processed further in regions higher up the ventral pathway.

The stimuli used to investigate curvature have been justifiably varied with configurations spanning highly controlled, narrow band stimuli (Dumoulin and Hess, 2007) to more naturally realistic images (Yue et al., 2020). Here, we use radial frequency (RF) patterns, which have been presented in studies of processing of shape curvature in humans (Wilkinson et al., 2000) and nonhuman primates (Tang et al., 2020). RF patterns have also been a mainstay of psychophysical literature that has shown exquisite sensitivity to (Wilkinson et al., 1998) and global processing of low RF stimuli (Hess et al., 1999; Jeffrey et al., 2002; Loffler et al., 2003; Bell et al., 2007b; Lawrence et al., 2016). We predicted therefore that response preferences to, and representations of, low RFs will emerge in the ventral visual pathway.

## Materials and Methods

### Rationale

**Figure 1.**
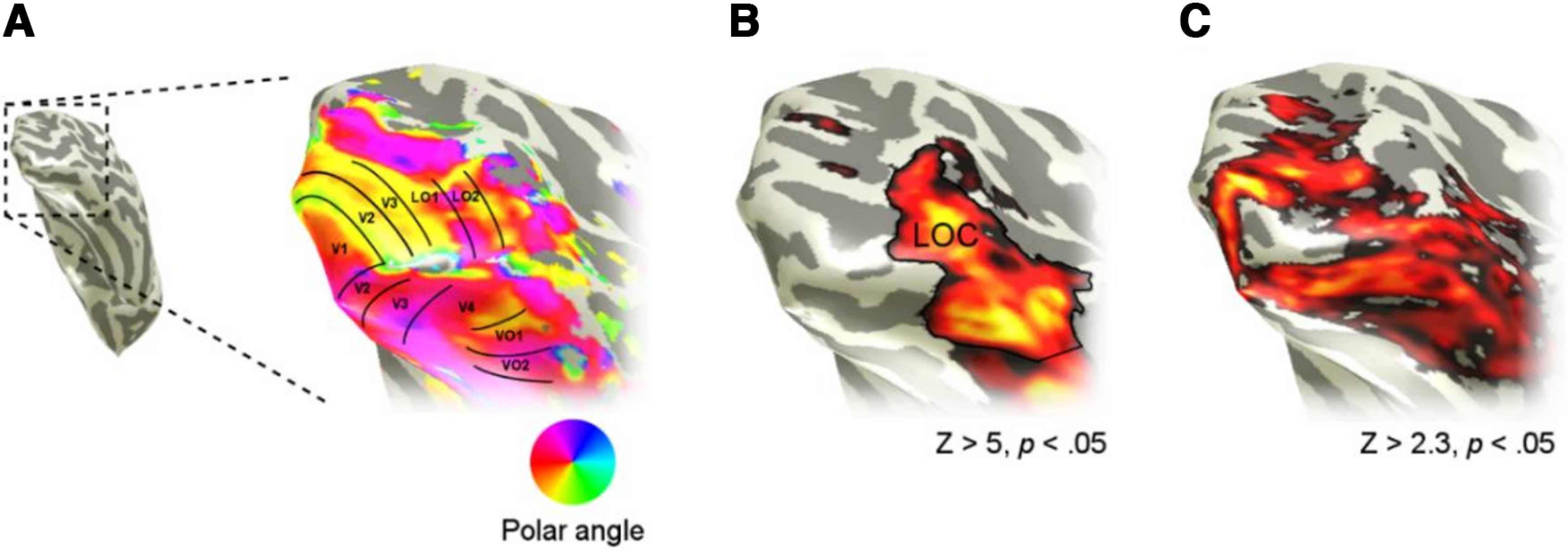
Typical data for an individual participant from the retinotopic mapping, LOC localizer, and stimulus localizer experiments for a single subject. ***A***, Retinotopic mapping was used to identify ROIs V1, V2, V3, LO1, LO2, V4, VO1, and VO2. ***B***, An objects versus scrambled objects localizer was used to identify LOC as a cluster of object-selective voxels in lateral occipital cortex. We analyzed only the posterior aspect of LOC, which we refer to as LO. ***C***, A stimulus localizer experiment was used to identify voxels in visual cortex that responded to the stimuli. All ROIs were constrained to only include these voxels to ensure that all analyses only considered voxels within the stimulus representation.

The aim of the study was to understand the tuning to RF in the following ROIs; V1-V4, LO1, LO2, and LO. While these regions together do not correspond to the complete ventral visual pathway, we predicted that global processing of shape would emerge in at least one of these regions. We followed a paradigm developed by Harvey et al. (2013) to allow tuning to a visual parameter, in their case numerosity and in our case RF, to be extracted from brain responses to a specific and effective stimulus regimen. The approach lends itself to in-depth assessment of relatively few individual participants, in our case eight, like the study by Harvey et al. (2013), by acquiring a large amount of data (∼5 h) from each individual. The paradigm is also well suited to establishing whether there are topographic mappings of the parameter under investigation. In our study, however, we found no strong evidence of a topographic mapping of RF. Our investigation therefore followed the general approach of evaluating univariate and multivariate responses within the ROIs that were identified in individuals from retinotopic mapping and functional localizer experiments to establish what RF tuning properties those regions exhibited (Fig. 1). In the sections that follow, the participants we tested are described followed by the MRI details, and then the procedures for retinotopic mapping, LOC localizer, and RF tuning experiments are given.

### Participants

Eight participants (mean age 28.00 years, SD 4.75 years; 5 males) were recruited from the University of York Psychology Department. All participants had normal or corrected-to-normal visual acuity, were naive to the aim of the study, and gave written informed consent. All participants completed 5.25 h of scanning in total, including a structural session, a retinotopic mapping session, an LOC localizer session, and two main RF tuning sessions. The study was approved by the York Neuroimaging Center Ethics Committee in accordance with the Declaration of Helsinki.

### MRI

#### fMRI data acquisition

All imaging data were acquired on a GE 3-Tesla Sigma HD Excite scanner using a 16-channel half-head coil to improve signal-to-noise in the occipital lobe. Acquisition parameters and analysis procedures for each session are described below. Across all experiments, stimuli presentation were controlled using MATLAB and Psychophysics toolbox (Brainard, 1997). Stimuli were presented using a projector and mirror setup (Dukane Image Pro 8942 LCD projector, pixel resolution 1280 × 1024, 60 Hz frame rate) at a viewing distance of 57 cm.

#### Structural scans

We acquired three, 16-channel, T1-weighted anatomic images (TR = 7.8 ms, TE = 3.0 ms, TI = 600 ms, voxel size = 1 × 1 × 1 mm^3^, flip angle = 12°, matrix size 256 × 256 × 176, FOV = 25.6 cm), one 8-channel T1-weighted anatomic image to aid alignments (TR = 7.8 ms, TE = 2.9 ms, TI = 450 ms, voxel size = 1.13 × 1.13 × 1 mm^3^, flip angle = 20°, matrix size 256 × 256 × 176, FOV = 29 cm), and one T2*-weighted fast gradient recalled echo scan (TR = 400 ms, TE = 4.3 ms, voxel size = 1 × 1 × 2 mm^3^, flip angle = 25°, matrix size 128 × 128, FOV = 26 cm).

T1-weighted anatomic data were used for coregistration and gray-white matter segmentation. To this end, the three 16-channel T1 scans were aligned and averaged together. We then divided this average by the T2*-weighted data to improve gray-white matter contrast and partially correct for the signal drop-off caused by use of a half-head coil. The resulting average T1 was then automatically segmented into gray and white matter using FreeSurfer (Fischl, 2012) and, where necessary, manually improved after visual inspection using ITKGray (https://web.stanford.edu/group/vista/cgi-bin/wiki/index.php/ItkGray).

### Retinotopic mapping

Each participant completed 6 wedge scans (size: 90°, rotating counterclockwise) and 2 expanding ring scans for this session (TR = 3000 ms, TE = 30 ms, voxel size = 2 × 2 × 2 mm^3^, flip angle = 90°, matrix size 96 × 96 × 39, FOV = 19.2 cm). Each scan contained 8 cycles of wedges/rings, with 36 s per cycle, traversing a circular region of radius 14.34 deg. Both wedges and rings were high contrast (>98%, 400 cdm^−2^) checkerboard stimuli that flickered at a rate of 6 Hz. Participants were instructed to attend to a central red fixation cross throughout the scans.

We performed standard analysis on the retinotopy data (Wandell et al., 2007), as specified previously (Baseler et al., 2011). For each participant, we identified V1-V3 as our early retinotopic regions, V4 for comparison with Macaque literature, and LO1/LO2 (Larsson and Heeger, 2006) as potential transitionary regions between retinotopic and object-based representations (Silson et al., 2013; Vernon et al., 2016). All ROIs were identified in both hemispheres for all participants; however, as we had no *a priori* reason to suspect hemispheric differences, all ROIs were collapsed across hemispheres.

### LOC localizer

To identify the LOC, each participant performed three 8 min localizer scans (imaging parameters were identical to those used for retinotopy). Each scan comprised 16 interleaved objects and scrambled objects blocks in an ABAB design format. Each block lasted 15 s, with one stimulus presented per second (0.8 s presentation, 0.2 s interstimulus interval). To ensure participants attended to the stimuli throughout the session, they performed a one back task in which there could be one, two, or no repeated items within a given block, while maintaining fixation at a red central cross. All stimuli were presented on a full screen mid-gray background (200 cdm^−2^), and there were no baseline/rest periods between blocks.

Stimuli comprised 225 easily recognizable grayscale object images. Background information was removed and image histogram was equalized. All objects were set to subtend 4 × 4 degrees visual angle on average. The scrambled object images were obtained by splitting the object images into squares of 0.8 × 0.8 degrees of visual angle in size. Any squares lying within the convex hull of the object were then randomly permuted and rotated. This removed any coherent form, while preserving both the coarse global shape profile and local details. Furthermore, a Gaussian filter (SD 1px) was applied to both object and scrambled image sets.

Localizer data were analyzed using FEAT (FMRI Expert Analysis Tool; Worsley, 2001). At the first (individual) level, we removed the first three volumes and used a high-pass filter cutoff point of 60 s to correct for low-frequency drift. Spatial smoothing was set to 4 mm and FILM prewhitening was used. Head movements were corrected for (MCFLIRT), and the resultant six motion parameters were entered as confound covariates in the GLM model. To combine data within participant, we ran fixed-effects analysis with cluster correction (*Z* > 5.0, *p* < 0.001).

Gray matter-restricted, cluster-corrected significant activity from the LOC localizer was rendered on the individual surface of each participant. LO was manually defined as the largest cluster in each hemisphere, avoiding overlap with the retinotopically identified nearby LO2 region. These clusters were then mapped back to gray matter, collapsed across hemispheres.

### Stimulus localizer

To restrict ROIs to the stimulus representation, participants performed a localizer scan (imaging parameters were identical to those used in the retinotopic mapping session). Each scan started with a 10 s fixation period, followed by 5 blocks of standard and 5 blocks of control RF stimuli (see RF stimuli), presented pseudo-randomly and interleaved with fixation blocks. In each block, the seven individual stimuli (RF2-RF7 and RF10 patterns) were presented twice, resulting in a 14 s block (stimuli were on screen for 0.8 s, followed by a 0.2 s interstimulus interval). Patterns were presented centrally against a mid-gray background, and participants were instructed to fixate on a black central cross while performing an oddball task on the RF stimuli (1 in 10 were contrast reversed).

Data analysis followed the description in the LOC localizer section, with the exception that 5 dummy volumes were removed to allow the scanner to reach a steady state, and the high-pass filter cutoff point was set to 84 s. We used cluster correction (*Z* > 3.1, *p* < 0.05) to identify significant voxels with which to restrict ROIs.

This localizer primarily affected V1-V3, keeping an average of 28.6%, 42.2%, and 52.2% of voxels, respectively. The V4 (89.1%), LO1 (96.3%), LO2 (85.1%), and LO (88.6%) ROIs were largely preserved.

### RF tuning

Each participant completed 2 sessions, each comprising eight, 294 s scans. In each session, we presented a stimulus set that comprised either a range of RF patterns, we term the “RF” stimulus set, or those same RF patterns with the addition of a high (rf = 20) ripple added to them, a set we term the “RF-ripple” stimulus set. The order of the sessions was counterbalanced across participants. Imaging acquisition parameters were identical to those from the retinotopic mapping session apart from the TR now being set to 2 rather than 3 s.

#### RF stimuli

RF patterns (Wilkinson et al., 1998) are defined using the following formula:

$$r\left(\theta \right)=r_{0}\left(1+A\left(sin\left(\omega \theta +\phi \right)\right)\right)$$

Theta (θ) represents the angles around a circle's perimeter, allowing the sinusoidal modulation of that perimeter by altering frequency (ω) and amplitude (A; set to 0.1), rotation can be set by altering phase (ϕ). The mean radius (r_0_), governing the average size of the stimulus, was set to 2.5° visual angle.

For the RF stimulus set, we used frequencies 2-7, 10, and 20. We also introduced an additional sinusoidal modulation set to an RF of 20 to patterns RF2-RF7 and RF10 (and RF20 was unchanged) to generate an alternative stimulus set, RF-ripple. The RF-ripple stimuli allowed the global shape of low RF patterns to be largely preserved but altered the contrast energy of these stimuli, so they were better matched to those with higher RF (see Stimulus properties).

To display the shapes, the contours were rendered against a mid-gray background using the fourth derivative of a Gaussian (Wilkinson et al., 1998) at 50% contrast, yielding a peak spatial frequency of 2 cycles per degree.

#### Stimulus properties

Previous work has shown that spatial frequency and contrast energy content of RF stimuli can vary with both RF and the amplitude. Moreover, variations in these stimulus properties captured a relatively large amount of the multivariate response in visual cortex (Salmela et al., 2016). In the present study, therefore, we attempted to account for and change the relationship between RF and contrast energy and largely equate spatial frequency content of our stimuli.

**Figure 2.**
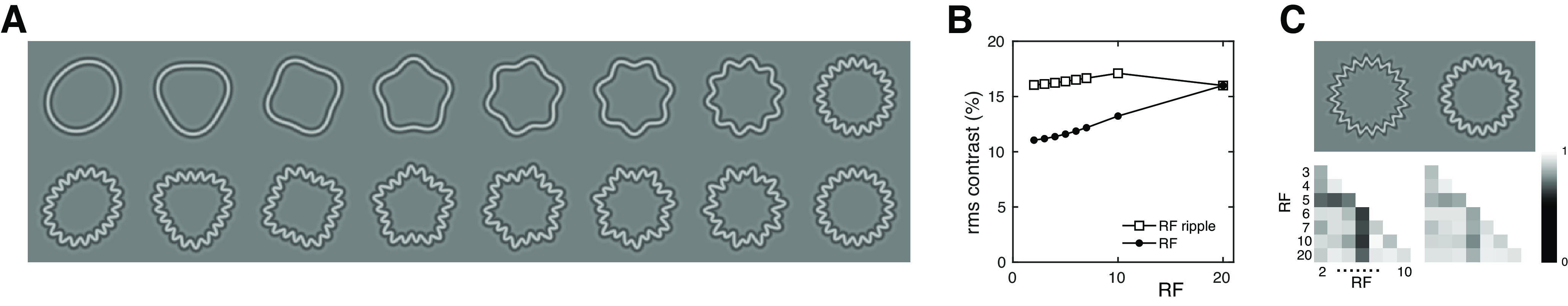
The RF stimuli and their contrast and spatial frequency properties. ***A***, RF stimuli presented to participants (top row) and their RF-ripple counterparts (bottom row). Exemplars are shown for a single orientation only. During the acquisition of BOLD responses, however, six orientations of each of the stimuli shown were presented. ***B***, The root-mean-square (rms) contrast of the stimuli computed as the mean over the annulus that capture all contrast variations of the six orientations presented. Filled circles represent RF stimuli. Open squares represent RF-ripple stimuli. ***C***, Top two panels represent different rendering approaches; the luminance profile is computed on the basis of the radial distance from the contour (left) or the perpendicular distance from the contour (right). The rendering techniques result in different spatial frequency variations between our stimuli as shown in the correlation between the two-dimensional amplitude spectra of stimuli at different RFs (bottom two panels). Moreover, radial rendering produces lower and more varied correlation between spatial frequency content of RF stimuli (bottom left) than the rendering derived from the perpendicular distance that was used in the present study (bottom right).

Figure 2*A* shows the stimuli we presented: RF stimuli and RF-ripple stimuli in the top and bottom rows, respectively. The contrast energy of RF stimuli increases monotonically with RF; but for the RF-ripple stimuli, it varies less and is no longer monotonic (Fig. 2*B*). Because many neurons respond to contrast and our stimuli exhibit variations in contrast, a consideration of how a contrast tuned response may register in terms of RF tuning is needed. For the RF stimulus set, a contrast tuned response would register tuning to high RF stimuli, while it would be tuned to a lower RF (∼10) for the RF-ripple stimuli. It is important to note that these tunings are to RFs that are greater than those that are processed globally (<7); and therefore, tunings to shape curvature that are behaviorally relevant can be disambiguated from tunings that are driven by sensitivity to contrast.

We also took a precaution to largely equate our stimuli for spatial frequency content. Spatial frequency content can change with amplitude and RF when the contrast profile of the stimulus is computed from the radial distance from the RF contour alone (Salmela et al., 2016). We used the distance perpendicular from the RF contour as the input to the fourth derivative function that defined the contrast profile of our stimuli. Our approach has the effect of all but removing and variation in spatial frequency content (Fig. 2*C*). The alternative rendering of the profile uses only the radial distance from the contour to compute the fourth derivative, which is entirely appropriate at threshold, but does introduce greater variability in spatial frequency content for suprathreshold stimuli that we use (Fig. 2*C*).

The approaches we took to control for contrast and spatial frequency content of stimuli allow us to investigate responses that are related to low RFs that are known to be processed globally and differentiate them from responses that are largely driven by variations in contrast energy and spatial frequency (Salmela et al., 2016). It should also be noted that orientation content of the stimuli scales with RF of the RF stimuli in much the same way as contrast energy but will again be largely equated in the RF-ripple stimuli, so we will refer to contrast energy and orientation content together when discussing the results.

#### Experimental design

Each RF tuning scan started with a 10 s fixation period, followed by two cycles of stimulus blocks. Each block lasted 6 s, during which each RF pattern was repeated 6 times (0.8 s presentation and 0.2 s interstimulus interval; phase of each pattern was randomly selected between the range of 0:60:300 degrees). The order in which the stimuli were presented followed the design used by Harvey et al. (2013): a ramp-up sequence, with RF2-RF7 and RF10, followed by 24 s of RF20 (baseline), and a ramp-down sequence (RF10, RF7-2) followed by another 24 s of RF20. Each scan terminated with 20 s of fixation to capture the full hemodynamic response for the final stimulus. The same procedure was used for both standard RF stimuli and RF-ripple stimuli. Participants performed the same oddball (contrast reversal) task as described for the stimulus localizer to ensure attention was maintained.

#### Modeling

The data were first preprocessed using FEAT: the first 5 volumes were removed to allow magnetization to reach a steady state, followed by high-pass filtering (cutoff 100 s), slice timing correction, and motion correction (MCFLIRT). No spatial smoothing was applied to the data, and all runs were coregistered to each participant's high-resolution structural space. We then extracted and concatenated the time series of all voxels from the restricted ROIs. Additionally, to create a null distribution for modeling, time series were extracted from 20,000 randomly selected voxels that were located in the white matter underlying the gray matter ROIs.

To model the data, we applied a Gaussian function to our experimental design, parameterized by a mean preferred RF plus a tuning width (SD). This was convolved with a standard double γ hemodynamic response function (hrf) to estimate the BOLD response we would expect based on the respective tuning parameters. We did not limit mean RF and its width to integers because each voxel will pool responses over a population of neurons exhibiting different tunings. We did also test logarithmic Gaussian models, but no improvements were found.

This Gaussian predictor was concatenated for all runs; we also included separate run-wise predictors for the temporal derivative (to allow slight temporal deviations across runs), a constant, a predictor for oddball events (again convolved with default hrf), and motion confound covariates. All run-wise predictors were set to zero outside their respective runs, and all predictors were high-pass filtered to match the data. After fitting these predictors to our data, we took the sum of squared residuals as our estimate of model accuracy.

To perform the fitting, for each voxel, we first tested ∼3500 initial models, with means ranging −2.5:20 (increments of 0.25) and tuning widths (SDs) ranging 0.5:20 (0.5 increments). The best fit for each voxel was then further refined using nonlinear least squares optimization (MATLAB's *lsqnonlin*). The fitting limits were set to −5 ≤ mean ≤ 30 and 0 < SD ≤ 30; constraining the mean preferred RF to be reasonably close to our stimulus range to enforce plausible fits.

The white matter voxel fits were used to generate a null distribution of fit accuracy; and from this, we calculated (two-tailed) significance for our fitted ROI voxels. We only kept ROI voxels that remained significant after correction for multiple comparisons (accounting for all included ROI voxels using Benjamin and Hochberg FDR correction). We also excluded voxels whose fitted parameters were within 0.1 units of our fitting limits (i.e., mean ≤ −4.9 or ≥ 29.9; SD ≤ 0.1 or ≥ 29.9), as we may not have found the best possible fit for such voxels; and even if we had, the interpretability of fits so close to the limits would be questionable.

**Figure 3.**
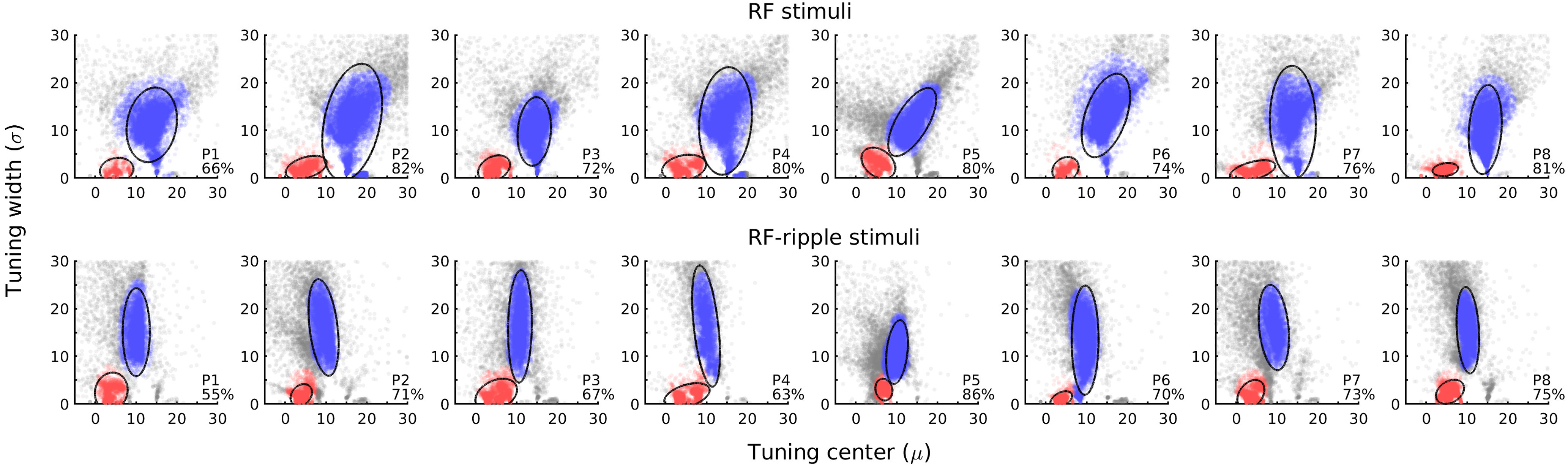
Model fits for each individual. Each panel plots the RF tuning center (µ) and bandwidth (σ) for every model that exceeded statistical thresholding. Top and bottom rows represent data for the RF and RF-ripple stimuli, respectively. The model parameters fall into two clusters shown in blue and red. Tuning to low RF, with small bandwidths (red), was evident in each participant and was largely unchanged for the stimulus set. Tuning to higher RF was also common to all participants (blue) but differed for the different stimulus sets. Inset in each panel, The percentage of the voxels identified in localizer scans that survived thresholding (the mean across participants was 76% and 70% for the RF and RF-ripple stimuli, respectively).

To assess the distribution of the fits across participants, we nonparametrically estimated the probability density functions of each ROI using kernel density estimation (*ksdensity* in MATLAB; normal kernel, default bandwidth, 200 × 200 equally spaced points encompassing fitting limits). We then graphically inspected the resulting distributions for each ROI via density plots. We preempt those results here to allow a fuller description of the methods we used to assess them quantitatively (below): two clusters emerged (see Figs. 2, 3) tuned to low and high RFs.

To further quantify and characterize the properties of the fits, we applied a clustering analysis using density-based spatial clustering (dbscan in MATLAB) to the model fit parameters derived from the whole brain. Both preferred mean (RF tuning) and SD (tuning width) were first standardized (*z* scores) to ensure neither dominated the clustering, and we specified a minimum of 500 voxels to form a cluster. The search radius (ε) was first estimated using a *k*-distance graph, then manually adjusted to best segment the observed clusters, while including as many voxels as possible. The preferred RF and corresponding tuning widths were then extracted for each resulting cluster. To inform on the distribution of RF tuning within each ROI, we calculated the proportion of each ROI's voxels that fell into a given cluster obtained from the whole-brain analysis approach.

#### Representational similarity analysis (RSA)

Similarity of neural representations between RF patterns in each ROI and across stimulus sets was tested using multivoxel pattern activity analysis. Specifically, voxel-wise patterns of activation for each session, ROI, and participant were extracted by first concatenating all scans within one session, and then building a corresponding GLM model. Here, each RF pattern, apart from RF20, which was treated as “baseline” common to both sessions, was entered as a regressor of interest. The relative temporal derivatives, together with confound regressors modeling the oddball events in each scan and the six FSL-generated motion regressors, were added to the model. After extracting activity patterns, we ran RSA by correlating the voxel-wise activity patterns for all pairwise combinations of RF patterns for each ROI and stimulus set. We then established predicted similarity matrices on the basis of RF differences between items in each stimulus set. Two stimulus predictors were used: one based on the log ratio of stimulus item pairs and the other on the difference in frequency of the pairs. The former prediction is a better approximation to how RF is perceived: unit differences between low RFs, such as 2 and 3, are perceived as far greater than the same differences between high RFs, such as 19 and 20.

Because the stimulus regimen was optimized for pRF methods (Harvey et al., 2013) and not for RSA, there was a potential for statistical dependence between responses to stimuli that neighbored each other in time, which in our case frequently meant neighboring RFs. We applied prewhitening (as specified above) to reduce statistical dependencies of this nature. However, separate to our RSA, we also used Leave One Out (LOO) cross validation to gain an insight to the reliability of patterns for identical stimuli, which are not as susceptible to statistical dependency because stimuli are not neighbors in time. In addition, we also extracted similarity matrices for white matter tissue (as specified above) to serve as a measure of the latent structure of the similarity that arises from residual statistical dependence and not stimulus-related responses. This similarity matrix was then used as a third predictor of the similarity matrices extracted from gray matter ROIs.

### Statistical analysis

The design of the study is repeated-measures with a relatively small number of participants examined in detail. We implement inferential statistical tests, most frequently ANOVAs, to examine effects of stimulus set and ROI on outcome measures. In the cases where interactions were observed, we followed up with appropriate *post hoc F* and *t* tests to examine specific effects. Data met assumptions for the tests, although sometimes only after appropriate transformation of the data to ensure equal variance assumptions were met. The raw and transformed data are presented in the manuscript. We also used correlation as a measure of relationships between variables and subjected them to inferential tests after appropriate Fisher transformation of the data to ensure normality. With a relatively small number of participants, the statistical tests used reached significance only when the vast majority, if not all, participants exhibited an effect, consistent with Type I errors being appropriately minimized.

## Results

To evaluate the ventral pathway's responses to, and representation of, RF patterns, we conducted the following analyses and report on them under different headings within Results. First, we examined the distribution of RF tuning and bandwidths across voxels that fell within all the ROIs we identified, and then separately for each ROI. Second, we performed a quantitative analysis of low and high RF tuning clusters that were evident in the distributions. This allowed us to examine the effects of two stimulus sets (RF and RF-ripple) and ROI on the tuning to RF. Third, we examined the representation of RF in the ventral pathway using RSA.

### The distribution of RF tuning and bandwidths

Our first assessment of the RF tuning properties was to scatter plot (Fig. 3) model parameters, the mean (µ) and SD (σ) for each participant collapsed over all ROIs. This serves two purposes: (1) it captures what the model characteristics are and whether they cluster; and (2) whether they vary across participants or between the stimulus sets (RF-stimuli and RF-ripple stimuli). The scatter plots show that models that survive statistical thresholding fall into two clusters in each participant: one in the bottom left of each plot (in red) corresponding to narrow tuning to low RFs, which are globally processed, and the other located more to the right in the plot (in blue) reflecting broad tuning to higher RFs. The location of the clusters in the scatter plots varies little across participants. Furthermore, the addition of the ripple to the stimuli shifts the cluster centered on high RFs to a lower RF, but the low RF tuned cluster appears largely unchanged. Because we have assessed responses from a relatively small number of participants in detail, it is important to demonstrate that the general characteristics of the signals we record are shared across all participants (as shown in Fig. 3). This feature has a strong bearing on inferential statistics that we present later, which are only likely to show significant effects if all participants of a small group share very similar response characteristics.

The ellipses shown in the scatter plots capture 95% of the models in each cluster, and we use the coordinates of center of the ellipses as a measure of the cluster's central tendency. We also captured the proportion of voxels in the ROIs that survived thresholding (Fig. 3, inset in each panel). The values are relatively high (55%-86%) given that the ROIs were defined by high powered block design experiments that captured responses to the annular region (compared with uniform gray) of the visual field where stimuli were presented in contrast to the stimulus regimen used to capture RF tuning in which the target stimuli were presented for the vast majority of the time.

**Figure 4.**
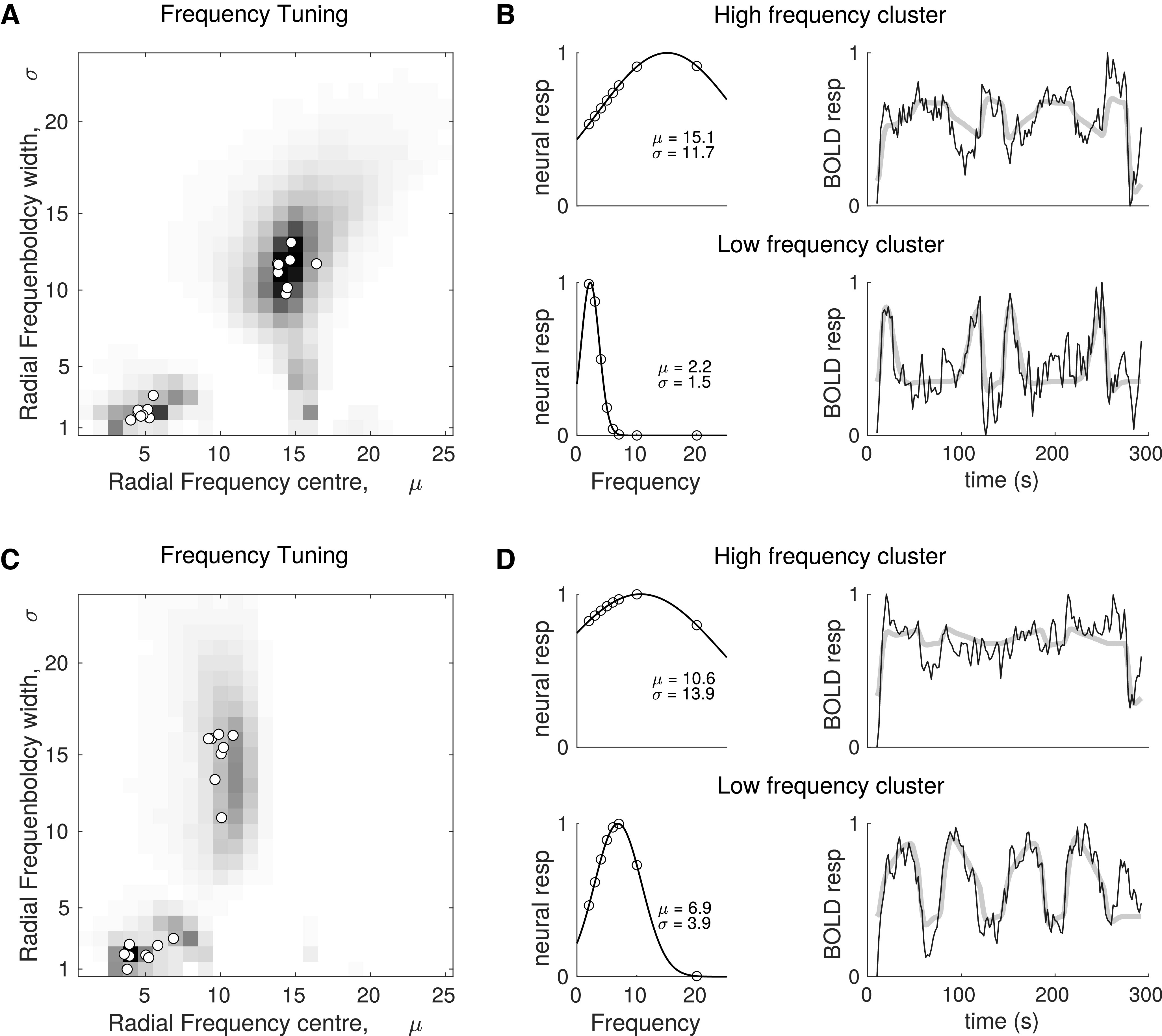
RF tuning properties of visual cortex. ***A***, ***C***, Grayscale renderings of a two-dimensional histogram of the modeled RF tuning of voxels in all ROIs (V1-V4, LO-1, LO-2, and LO) for the RF and RF-ripple stimuli, respectively. Modeled tunings were retained only if significant, which was the case for 70% (RF) and 68% (RF-ripple) of voxels in the stimulus representation. Open circles represent the central tendency of the individual plots (see Fig. 3). Tunings that are representative of the clusters found at low and high RF are shown in ***B***, ***D*** for the RF and RF-ripple stimuli, respectively. Left, The population neural response as a function of RF. Top and bottom graphs represent high- and low-frequency cluster tunings. Right, Examples of time series data (black) and the best fitting model (thick gray) derived from the population neural responses shown in the adjacent graphs. ***B***, ***D***, Top rows represent responses that capture contrast and orientation content of the stimuli as shown in Figure 2, with a largely monotonic increase in response as a function of RF for the RF stimuli, but not for the RF-ripple stimuli. ***B***, ***D***, Bottom rows represent tunings to different RFs, 2.2 and 6.9, to illustrate the range of tunings observed in the low-frequency cluster common to both the RF and RF-ripple stimuli.

The data shown in Figure 3 are aggregated in a two-dimensional histogram of the RF tuning and bandwidths (Fig. 4*A*,*C*). As expected from the individual data, two clusters are evident: one in the bottom left of the histograms, which corresponds to a narrowband tuning at low RFs; and the other more centrally located in the histogram and corresponding to broadband tuning to higher RFs. Superimposed on the histogram are the cluster centers for each participant, which demonstrate narrow dispersion of this measure between participants.

Examples of the RF tuning functions and the way that they model the BOLD time series are shown in Figure 4*B*, *D*. For the RF stimuli, the representative tuning to the high RF (µ = 15.1), which is also relatively broadband (σ = 11.7), exhibits a broadly monotonic increase in response with increasing RF (Fig. 4*B*, left, top), similar to the contrast energy of the stimuli that are shown in Figure 2*B*. For the RF-ripple stimuli (Fig. 4*D*, left, top), the representative tuning to high RF is centered on a lower RF (µ = 10.6) and has a higher bandwidth (σ = 13.9) than for the RF stimuli. The tuning function (Fig. 4*D*, left, top) no longer has the monotonic relationship between RF, and there are smaller variations in response as a function of RF overall, again capturing quite well the relationship between contrast energy and RF of the RF-ripple stimuli shown in Figure 2*B*. The tunings to high RF appear therefore to be common and are consistent with responses driven by contrast and likely orientation rather than global shape properties.

The tunings to low RFs (Fig. 4*B*,*D*, bottom left) do not exhibit response profiles that fit variations in contrast energy or orientation content of the stimuli for either the standard RF of RF-ripple stimuli. Moreover, the highest contrast stimuli are nonpreferred stimuli for these tuning functions. As a result, the best fitting models derived from the tunings to low RF differ markedly from those derived from the high RF tunings (Fig. 4*B*,*D*, graphs to the right in the top and bottom rows). The low-frequency tuning clusters for both the RF and RF-ripple stimuli cover the range of RFs that are processed globally; ∼2-7. We show selected time series and model fits for RFs near the limits of this range, namely, 2.2 in Figure 4*B* and 6.9 in Figure 4*D*, to capture how the time series reflect different (2.2 vs 6.9) low-frequency tunings.

**Figure 5.**
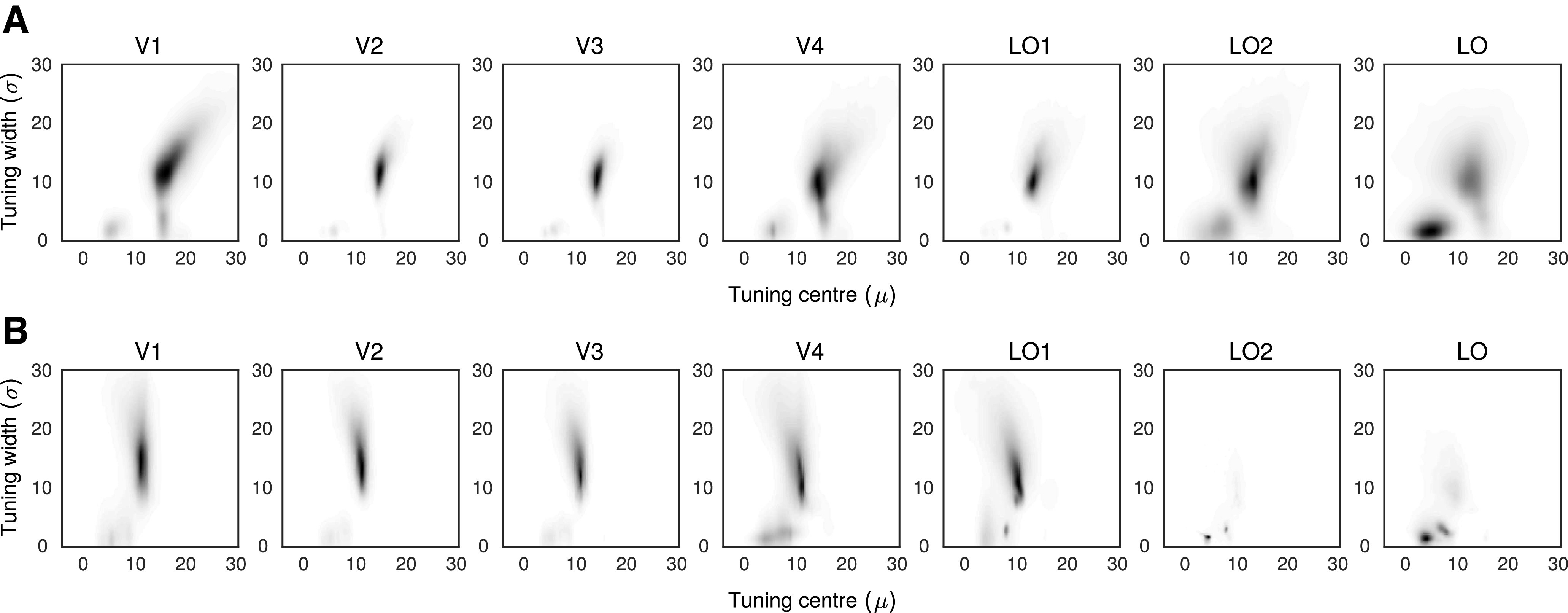
***A***, ***B***, RF tuning for ROIs. The model fitted parameters, preferred RF tuning and tuning width, mapped onto two dimensions. The grayscale maps the relative number of model fits. Darker colors represent more voxels. Two clusters of model fits emerged: one with broad tuning to high RF that dominated model fits in early visual areas and another narrowly tuned to low RF that emerged in V4 and are prominent in lateral occipital areas.

The next step was to assess the tuning preferences of each ROI (averaging across participants). We did this by computing probability density functions using kernel density estimation. This approach is broadly analogous to a smoothed 2D histogram and makes no assumptions about the underlying distribution of the data, allowing for further examination of the RF tuning distributions for different ROIs and for the RF and RF-ripple stimuli (Fig. 5). Predictably, this approach revealed the same two general tuning clusters that were detected when data were collapsed across ROIs (Figs. 3, 4). However, the high- and low-frequency tuning clusters occurred to different extents in different ROIs. The high-frequency tuning dominated in early visual cortex (V1-V3), while the low-frequency tuning became increasingly prominent up the visual hierarchy and dominated in LO. There were also understandable differences between the stimulus conditions. For the RF-ripple stimuli, the tuning to high frequencies was less common overall and largely absent in LO-2 and LO. In all regions exhibiting the high-frequency tuning, it was also centered on lower frequency for the RF-ripple than the RF stimuli as previously noted. The tuning to low RFs appears less affected by the change from RF to RF-ripple stimuli and is therefore more consistent with mechanisms processing global shape properties.

### Quantitative analysis of low and high RF tuning clusters

The visualization of the data above was for the mean data across all participants. To isolate the two clusters for each individual, we took all fitted voxels (initially agnostic of ROI) per stimulus set and participant and clustered them. This was straightforward for RF stimuli as the two clusters were well separated, while clusters were separated less for the RF-ripple stimuli; nonetheless, the clustering results showed notable consistency across participants (as demonstrated by 95% CIs below).

We found the “high-RF” cluster shifted and broadened across stimulus conditions as can be seen in Figures 3 and 4. Specifically, the mean preferred RFs in the RF and RF-ripple experiments were 14.50 and 9.90 (95% CIs: 13.92-15.09; 9.95-10.26, respectively), and tuning widths were 11.41 and 14.94 (95% CIs: 10.68-12.15; 13.62-16.26, respectively). This cluster appears to encompass voxels that have no specific preference to RF, with a bias toward higher-frequency stimuli, likely because of their greater contrast energy and orientation content. As such, the leftward shift and broadened tunings for RF-ripple versus standard RF stimuli make sense; the RF-ripple stimuli had higher contrast energy and orientation content (introduced by the RF 20 ripple) that varied less across the stimulus set, and so voxels that previously preferred (e.g., RF20) would now be expected to respond more equally across the stimulus range.

The second “low-RF” cluster was almost identical across stimulus sets; the mean preferred RFs for the RF and RF-ripple experiments were 4.80 and 4.76 (95% CIs: 4.46-5.13; 3.95-5.57, respectively), and tuning widths were 2.03 and 2.08 (95% CIs: 1.68-2.37; 1.65-2.51, respectively). The comparable tunings across both sessions suggest that there is a subset of voxels that are sensitive to shape, defined by RF changes, in a more global sense, rather than sensitivity to local contour information alone. Given that this cluster analysis was performed over all ROIs, we further mapped the clusters to the ROIs to determine where the voxels that had a preference for the low and high RFs could be found.

**Figure 6.**
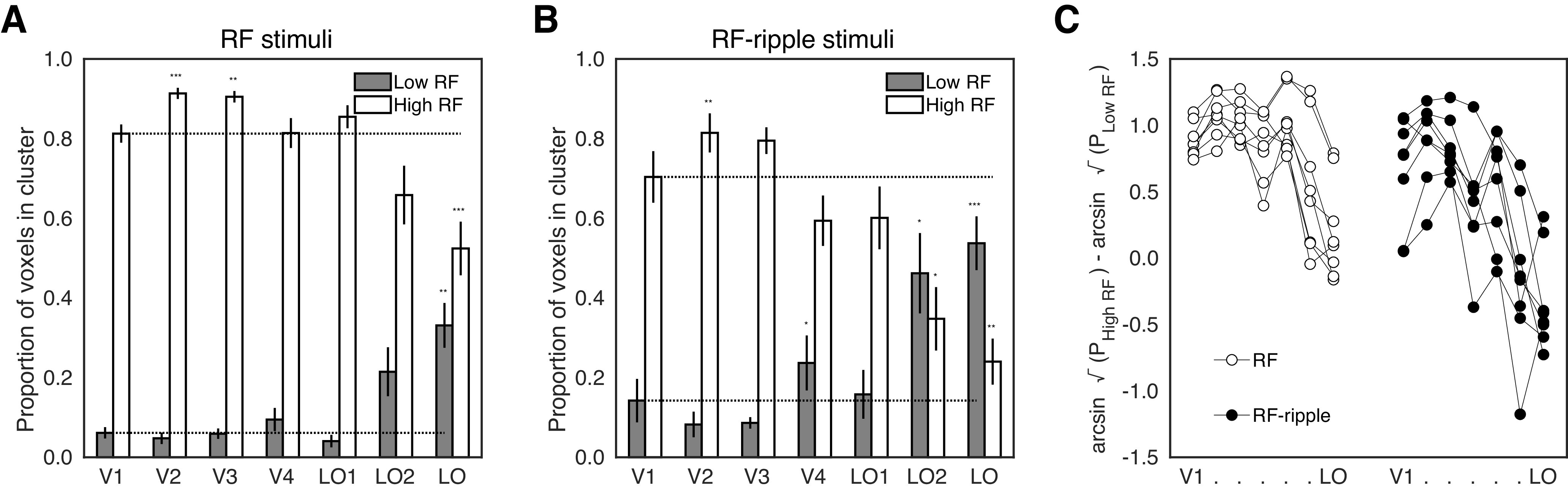
The proportion of model fits that fell into high (light bars) and low (dark bars) RF clusters. ***A***, Data are shown for the RF stimuli. ***B***, Equivalent data for the RF-ripple stimuli. Significant differences from V1 proportions: *p < 0.05, **p < 0.01, ***p < 0.001, as computed from planned contrasts. ***C***, The data in ***A***, ***B*** were submitted to a three-way ANOVA after transforming the data to ensure they met the assumptions of equal variance. Transformed data are plotted to capture the difference between voxels tuned to high and low RFs for each participant across all ROIs and the two stimulus sets, which underpins the three-way interaction reported in the text.

To map the two clusters (low-RF, high-RF) from each stimulus set to individual ROIs, we calculated the proportion of each ROI's voxels that fell into a given cluster. We assessed proportions for both clusters as voxels could be outside the two clusters (i.e., classed as “noise”; see gray data points in the scatter plots shown in Fig. 3); so if an ROI had more voxels in one cluster, it did not necessarily imply a proportional decrease in the other. The proportions of voxels falling into the low and high clusters are bar charted in Figure 6*A*, *B* for the RF and RF-ripple stimuli, respectively. In both charts, the proportion of voxels showing tuning to low frequencies increases up the visual hierarchy and in LO dominates for the RF-ripple stimuli.

The proportions were arcsine transformed (sin^−1^√p) to meet normality requirement for a 2 × 2 × 7 (Stimulus × Cluster × ROI) repeated-measures ANOVA, which was applied to the data. This resulted in a significant three-way interaction (*F*_(6,42)_ = 5.51, *p* = 2.8 × 10^−4^), which we help illustrate in Figure 6*C*, where the difference between the transformed proportion of voxels tuned to high and low frequencies is given for each individual for each ROI. Evident in each participant's data in the plot is the reduced difference between high- and low-frequency tuned voxels up the visual hierarchy and even greater reduction in this difference for the RF-ripple stimuli. Subsequent “Cluster × ROI” ANOVAs also yielded significant interactions between the two factors (for both stimulus sets, *p* < 0.001). To follow-up on the interactions, we ran four one-way repeated-measures ANOVAs exploring the main effects of ROI separately per stimulus condition and cluster.

ROI had a significant effect on the proportion of voxels mapped onto each cluster tuned to high or low RF patterns for both RF and RF-ripple stimuli (RF high/low: *F*_(2.20,15.37)_ = 24.12, *p* = 1.3 × 10^−5^; *F*_(2.25,15.77)_ = 12.90, *p* = 3.5 × 10^−4^, respectively; RF-ripple high/low: *F*_(2.44,17.11)_ = 23.98, *p* = 5.1 × 10^−6^; *F*_(1.80,12.59)_ = 13.54, *p* = 0.001, respectively). While this was informative, suggesting that it is possible to differentiate a preference in processing local (high RF) versus global (low RF) shape information, it did not allow us to identify where RF tuning preferences began to diverge from low-level representations. To this end, we ran planned comparisons, with V1 as “baseline” where, on average, 64.63% of voxels were in the high RF cluster, compared with 13.67% in the low RF cluster when the RF stimuli set was used. Similarly, in the RF-ripple session, 57.83% and 19.85% of voxels were mapped to the high and low RF clusters, respectively.

First, for the high radial cluster, in RF experiment, we found that V2 and V3 had significantly more high RF tuned voxels compared with V1 (V2: 73.35%, *p* = 3.8 × 10^−4^; V3: 72.47%, *p* = 0.005), whereas only LO had significantly fewer high RF tuned voxels (46.61%, *p* = 2.9 × 10^−4^). In the RF-ripple experiment, both LO2 and LO had significantly fewer high RF tuned voxels (LO2: 42.57%, *p* = 0.012; LO: 47.11%, *p* = 0.001), and only V2 had significantly more (65.58%, *p* = 0.007). No other results were significant (all *p* > 0.090).

For the low RF cluster, LO had significantly more low RF tuned voxels compared with V1 across both experiments (RF experiment: 34.36%, *p* = 0.001; RF-ripple experiment: 47.11%, *p* = 1.4 × 10^−4^). In addition, in the RF-ripple experiment, we also found greater proportions of low RF tuned voxels in V4 and LO2 (V4: 27.57%, *p* = 0.019; LO2: 42.57%, *p* = 0.024). No other results were significant (all *p* > 0.062).

In summary, as highlighted earlier, tunings for high RFs dominate in early visual cortex (V1-V3). Conversely, LO consistently diverged from V1, by showing the greatest preference for low RF patterns. However, V4 and LO2 did show differences from V1 for the RF-ripple experiment when variations in low-level contrast and orientation content in the stimuli were much reduced.

### Representation of RF in the ventral pathway

One consequence of LO's low RF tuning preference is that we would expect its pattern of activity to show consistency across the RF and RF-ripple stimuli, as both sets of stimuli share comparable global profiles. Conversely, for an ROI such as V1, which is likely responding to the contrast energy and orientation of our stimuli, we would expect different patterns of activation for the two sets of stimuli.

**Figure 7.**
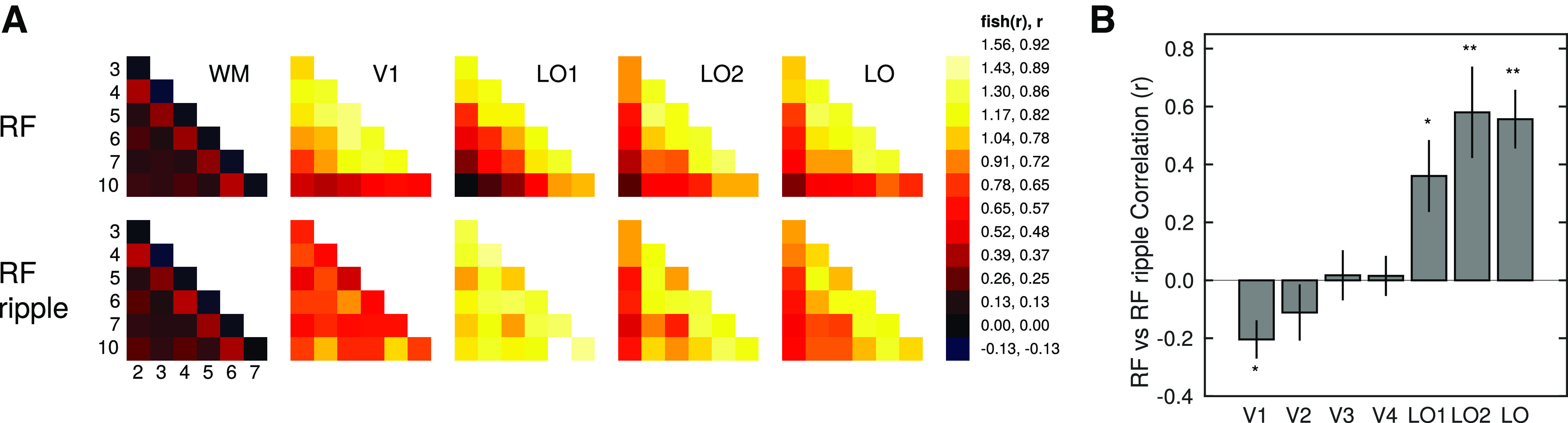
Similarity of responses to RF patterns across visual cortex. ***A***, Matrices of correlation between responses to RF patterns displayed as heat maps. Data are shown for white matter (WM) and ROIs V1, LO-1, LO-2, and LO. Top and bottom rows represent correlations for the RF and RF-ripple stimuli, respectively. ***B***, The correlation between the similarity matrices obtained from responses to RF (top row matrices) and RF-ripple (bottom row matrices) stimuli for each ROI. *p < 0.05. **p < 0.01.

To test this, we used multivoxel pattern activity to extract the voxel-wise patterns of activation related to each stimulus pattern for each stimulus set, ROI, and participant. RSA was then computed by correlating the voxel-wise activity patterns for all pairwise combinations of RF patterns (for each ROI per stimulus set). This generated similarity matrices, capturing neural similarity across RF patterns, which are shown in Figure 7*A* for V1 and lateral occipital ROIs. For the RF stimulus set, the similarity in patterns of activation is relatively high across all regions. For the RF-ripple stimulus set, large changes in similarity are observed for V1 (and other ROIs, V1-V4). In lateral occipital ROIs, similarity is better preserved from LO-1 through to LO. In LO, for example, the similarity matrix for RF and RF-ripple stimuli is largely unchanged (Fig. 7*A*). In addition, to assess how temporal correlations could result in statistical dependencies, we also computed similarity matrices for a white matter ROI, which show low correlations overall; and although there is some structure to the similarity matrices, it does not appear to follow the structure found in gray matter ROIs (Fig. 7*A*).

To test statistically how well the similarity was preserved across stimulus conditions, for each participant's gray matter ROI, we correlated that ROI's similarity matrix for the RF stimuli (Fig. 7*A*, top row) with its corresponding RF-ripple similarity matrix (Fig. 7, bottom row) and ran one-sample *t* tests on the (Fisher *Z*-transformed) results (Fig. 7*B*). We found that V1 had a significant negative correlation (*t*_(7)_ = −3.55, *p* = 0.009), but only LO1, LO2, and LO showed significant positive correlations (*t*_(7)_ = 2.61, 4.27, 5.98, *p* = 0.035, 0.004, 0.001, respectively). No other results were significant (all *p* > 0.305).

**Figure 8.**
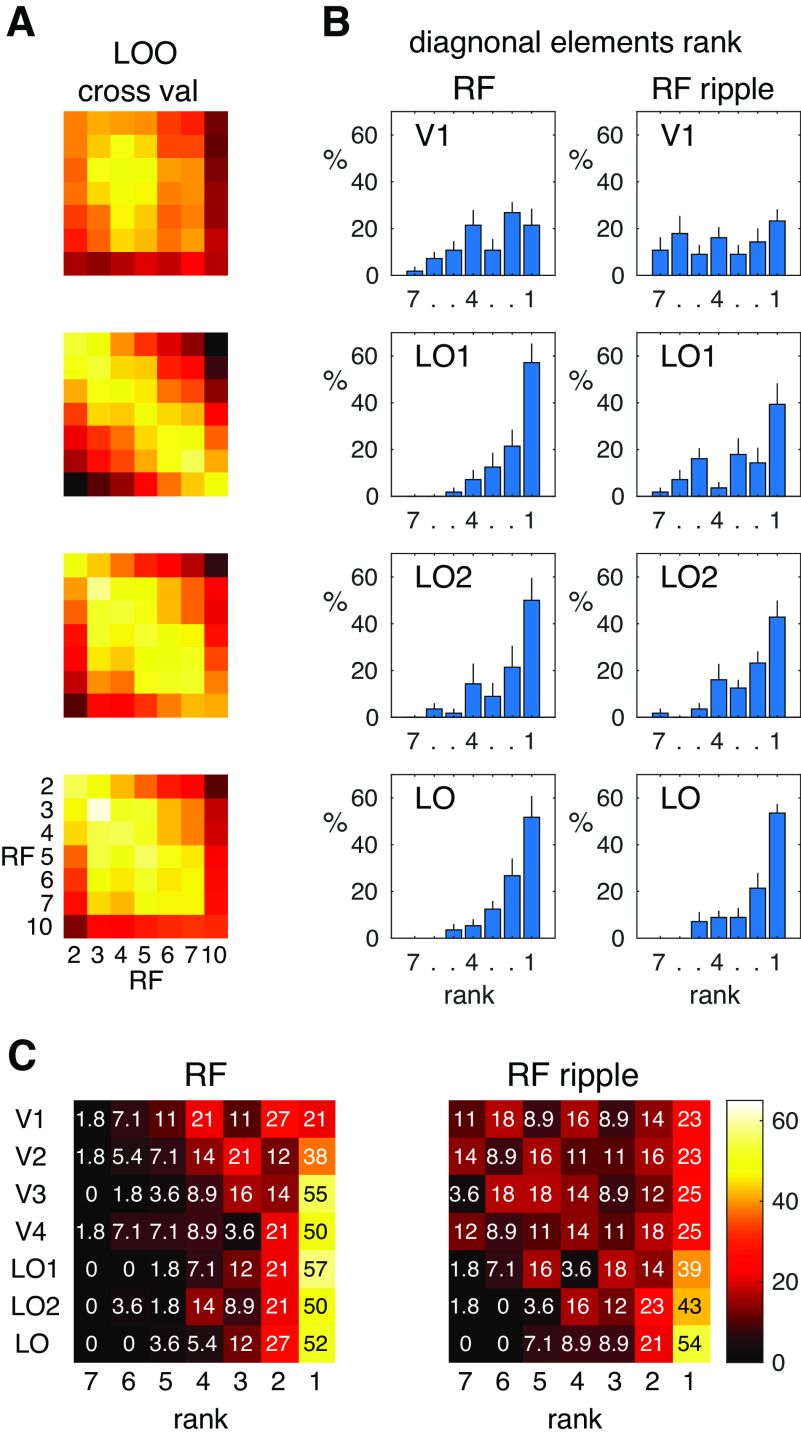
Cross validation of response similarity. ***A***, LOO cross validation of response similarities (as measured by correlation) for selected ROIs. Similarity matrices are shown for the ROIs V1, LO1, LO2, and LO (top to bottom) in false color (hot) rendered images. The diagonal elements largely exhibit the highest correlations. ***B***, Ranks (1 highest and 7 lowest) of the correlations for diagonal elements with respect to the mean of elements paired in the same row and column of the diagonal element (a total of six pairings in addition to the diagonal yielding ranks from 1 to 7). Frequency of the ranks (in %) was computed for each ROI and each participant; and mean (across participants) frequencies of ranks (and standard errors) are given in each panel. Data for the same ROIs (V1, LO1, LO2, LO) are given for the RF and RF-ripple stimuli in the left and right columns, respectively. In general, the diagonal elements commonly had the highest correlations and were therefore ranked 1. This gives a strong indication that statistical dependencies in the data are not a widespread contributor to the response similarities we measured (as presented here and in Fig. 7). ***C***, For completeness, the frequency (in %) of ranks is given for the diagonal elements in all ROIs in a matrix grid with false color (hot) background coding the percentages. Data are given for means across participants.

While it is reassuring to observe that our stimulus manipulation (RF vs RF-ripple) changes the similarity matrices selectively across ROIs, we also need to examine whether statistical dependencies that result from the stimulus regimen optimized for pRF analyses might explain some of the structure in the similarity matrices. Here, we present similarity matrices again, but now computed with cross validation using the LOO approach (Fig. 8*A*). This yields matrices that capture correlations between identical stimuli (the diagonal elements) for which statistical dependence is much less than for stimuli that are presented next to each other in time. If statistical dependence were driving the similarity, diagonal elements would exhibit low correlations relative to those not on the diagonal. This does not appear to be the case, however, in the matrices shown (Fig. 8*A*). To examine the diagonal elements still further, we computed their rank compared with mean of correlations in the same row and column. These are plotted in frequency histograms in Figure 8*B*, where it is evident that, for the lateral occipital ROIs, the majority of the correlations were highest (Rank 1) for the diagonal elements. In V1, this is not the case. We show in Figure 8*C* the ranks for all ROIs and this indicates that, although correlations for the diagonals are often high (as shown by the high frequency of Rank 1) for all ROIs, it is only for the lateral occipital regions that they are consistently high across stimulus conditions. It is possible, therefore, that, in early ROIs, statistical dependence may play a role in the structure of the similarity matrices. Therefore, we take this into account in our attempts to predict what is driving the structure of the similarity matrices we originally computed.

**Figure 9.**
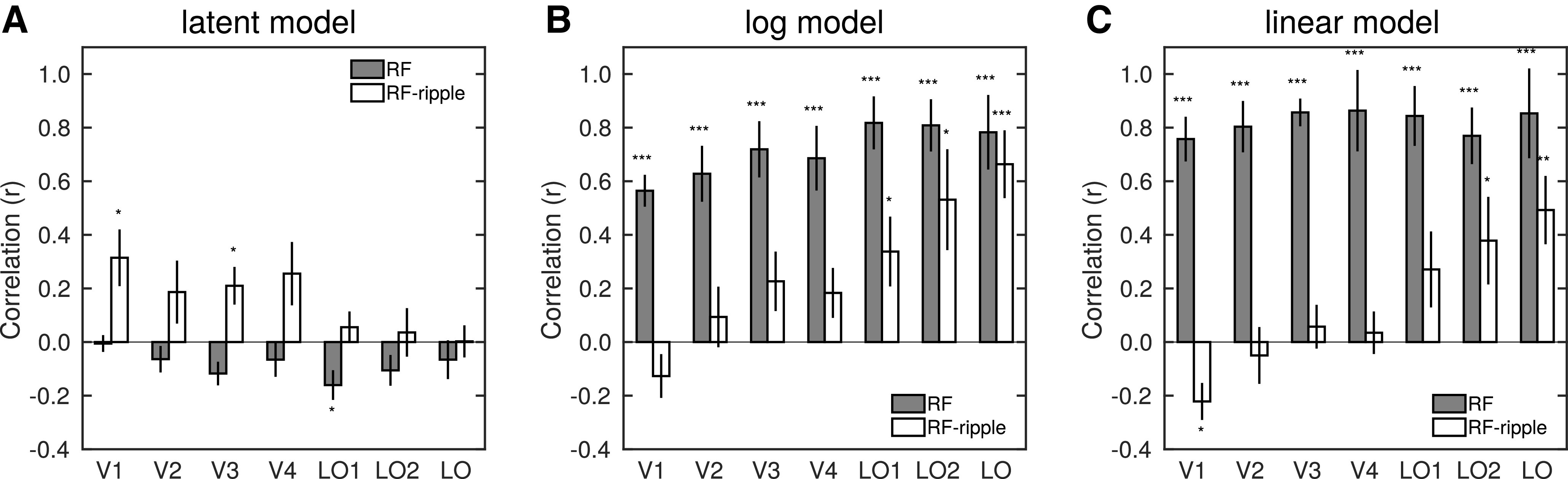
Predictors of response similarity matrices. ***A***, Bar plot of the correlations between the white matter ROI response similarity matrix and the response similarity in all gray matter ROIs. The white matter similarity matrix gives a measure of the latent structure that could arise from statistical dependencies in the data. ***B***, Correlations between stimulus similarity, as measured by the log difference between the RF of stimuli, and response similarity. ***C***, Correlations between stimulus similarity, as measured by the linear difference between the RF of stimuli, and response similarity. Data are given for the two stimulus sets, RF and RF-ripple, shown by gray and white bars, respectively. Distributions of the correlations are significantly different from zero: *p < 0.05, **p < 0.01, ***p < 0.001. Error bars indicate SEM.

Before embarking on predicting the similarity matrices on the basis of stimulus properties below, we fist examine whether the similarity matrices for white matter (as previously shown in Fig. 7*A*), which we refer to as the latent model, predict the similarity matrices in gray matter ROIs. In short, the structure in the white matter matrices is generally a poor predictor of the structure in gray matter matrices, with uniformly low or negative correlations for the RF stimuli and only significantly positive correlations for V1 and V3 for the RF-ripple stimuli (Fig. 9*A*). Importantly, for regions in lateral occipital cortex no significant positive correlations were observed, meaning we can rule out statistical dependence explaining the structure in their similarity matrices.

To test whether the relationships between patterns of response to RF stimuli were related to differences in the RF of the stimuli we presented, we created two predictor similarity matrices. The first used the log ratio of the RF of pairs of RF patterns (e.g., for RF2 and RF3; -abs(ln(2/3)) = −0.41). We used log ratios as we reasoned that the jump from e.g., RF2 to RF3 perceptibly much greater than the jump from e.g., RF20 to RF21, and so the similarity matrix should capture this. The second predictor matrix simply computed the difference in RF of the stimuli. This predictor might better capture variations in contrast and orientation, which were relatively large for the RF stimulus set and scaled with RF. The predictor similarity matrices were then correlated with all neural similarity matrices across ROI and stimuli (Fig. 9*B*,*C*).

Data for the log model predictions are given in Figure 9*B*, and the extent to which the model predicts the similarity of neural responses was assessed by computing single-sample *t* tests. The similarity of neural responses is predicted very well by the log model for the RF stimulus set (*p* < 0.001 for all ROIs); but for RF-ripple stimuli, the log model captures similarity of the neural responses only in LO1, LO2, and LO (*t*_(7)_ = 2.685, 3.10, 6.29, *p* = 0.031, 0.017, <0.001, respectively). No other results were significant (all *p* > 0.077). Data for the linear model are presented in Figure 9*C* and were assessed in the same way. Again, the similarity of neural responses to RF stimuli was predicted well in all ROIs (*p* < 0.001); while for the RF-ripple stimuli, the linear model significantly captured the similarity of neural responses only in LO2 and LO (*t*_(7)_ = 2.42, 4.21, *p* = 0.046, 0.004, respectively). There was also a significant negative correlation between the linear model's predictions and the similarity of neural responses in V1. No other results were significant (all *p* > 0.092). While the alternative models appear to have similar predictive power, it is noteworthy that the linear model has higher correlation than the log model with similarity of neural response to the RF stimuli. In contrast, for the RF-ripple stimuli, it is the log model that performs better, at least for some ROIs.

We assessed the two models' predictive power as a function of stimulus set and ROI by performing a three-way ANOVA of the Fisher-transformed correlation data. We acknowledge that caution must be taken in interpreting such an analysis, particularly for ROIs in which the models have low correlations with the similarity of neural responses. The three-way ANOVA showed a three-way interaction (*F* = 4.194, *p* = 0.002, η^2^ = 0.375), meaning that the extent to which the models fitted the data varied with stimulus type in a different way across ROIs. Also evident was the significant two-way interaction between the model and the stimulus type (*F* = 39.583, *p* < 0.001, η^2^ = 0.850) likely driven by the observation that the linear model worked best for the RF stimuli while the log model accounted better for pattern of responses to the RF-ripple stimuli.

In Table 1, we show the results of the follow-up analysis in which we ran two-way ANOVAs in each ROI and paired *t* tests. Two-way interactions were evident in V1 to V4 and LO. In V1 and V2, the interaction was driven by a better fit of the linear than the log model for the RF stimuli and an absence of differences in model fit for the RF-ripple stimuli, where both models performed very poorly. In V3 and V4, a similar advantage of the linear model was evident for the RF stimuli, but now the log model worked better than the linear model for the RF-ripple stimuli. While it is tempting to take the latter at face value, the correlations are low and straddle zero across participants; so although statistically significant, this increase in model fit may not be meaningful. In LO, the interaction was driven by significantly better performance of the log model over the linear model for RF-ripple stimuli and largely similar performance of the models for RF stimuli. This pattern of model fits is the opposite of that observed in V1. It is also important that, in LO, all model fit correlations were significantly different from zero, allowing the improved fit of one model over the other to be taken as meaningful.

**Table 1.** Results of two-way ANOVA applied to each ROI*^a^*

ROI	Model × stimulus (*F*, *p*, η^2^)	Model (*F*, *p*, η^2^)	Stimulus (*F*, *p*, η^2^)	RF: lin – log (*T*, *p*)	RF-ripple: lin – log (*T*, *p*)
V1	13.906, 0.007, 0.665	56.539, <0.001, 0.890	191.976, <0.001, 0.965	4.932, 0.002	−1.870, 0.104
V2	13.877, 0.007, 0.665	10.338, 0.015, 0.596	100.267, <0.001, 0.935	4.455, 0.003	−2.103, 0.084
V3	38.626. <0.001, 0.847	3.098, 0.122, 0.307	88.049, <0.001, 0.926	4.237, 0.004	−3.377, 0.012
V4	44.253, <0.001, 0.863	10.414, 0.015, 0.598	31.541, 0.001, 0.818	5.838, 0.001	−2.894, 0.023
LO1	3.389, 0.108, 0.326	0.002, 0.965, 0.000	50.855, <0.001, 0.879	0.602, 0.566	−0.901, 0.398
LO2	2.006, 0.200, 0.223	1.970, 0.203, 0.220	16.915, 0.005, 0.707	−0.828, 0.435	−2.062, 0.078
LO	8.540, 0.022, 0.550	0.066, 0.804, 0.009	8.552, 0.022, 0.550	1.459, 0.188	−3.445, 0.011

*^a^*ROIs are given in each row. Columns show data for the interaction (model and stimulus), main effects of model (linear vs log), and stimulus (RF vs RF-ripple). Post hoc *t* tests are for differences in the models for each stimulus type.

Overall, therefore, the multivoxel pattern activity results largely mirror the univariate findings, highlighting a move away from local toward more global or holistic processing that becomes clear and likely dominant in the lateral occipital cortex. Global processing of shape also appears in retinotopically defined LO1 and LO2. Together, the results reflect a processing pathway of shapes that moves from a low-level retinotopic representation toward a more abstract representation in anterior lateral occipital cortex.

## Discussion

We found two characteristic tuning profiles to RF stimuli. The tuning most commonly detected in the visual cortex was broad band and centered at relatively high RFs for which there is no behavioral evidence of global processing. Our stimulus manipulation that added a high RF component to the RF patterns shifted the tuning's center, indicating that low-level stimulus features that include contrast and orientation content were driving this aspect of the cortical response. The second tuning we detected was centered on the low RFs, for which there is strong behavioral evidence of global processing (Hess et al., 1999; Jeffrey et al., 2002; Loffler et al., 2003), and had a narrow bandwidth. This tuning remained, when the high RF ripple was added to our stimuli, which we, in common with others (Bell et al., 2007a), take as an indicator that the global properties of the shape were driving this aspect of the cortical response. We found that the low RF tuned responses were most evident in the extrastriate cortex, particularly in LO, but the high-frequency ripple added to stimuli (to render them less varied in orientation and contrast content) highlighted that this tuning to global shape could also be revealed in retinotopically defined areas LO1, LO2, and V4. Multivariate approaches showed that all the subdivisions of visual cortex we examined exhibited a relatively high level of similarity in the patterns of response across RFs, but that this pattern was disrupted very much in primary visual cortex and other early retinotopic areas, when the high RF ripple was added to stimuli. Indeed, the only areas that maintained the similarity across RF representations once the ripple was added were LO1, LO2, and LO. Finally, the similarity of the stimuli as quantified by the difference of the log RF provided a good prediction of how the pattern of responses was similar, consistent with human sensitivity to the global shape of RF stimuli.

### Stimulus properties that drive responses in early visual cortex

The responses of early visual cortex to RF stimuli revealed a strong bias to stimuli that exhibited the greatest contrast and orientation content. Given that contrast and orientation increased systematically with increasing RF of the RF stimulus set, the bias to contrast and orientation was expressed as a tuning of responses to high RF. This tuning was prevalent and dominated in early visual cortex. Our findings are consistent with early visual cortex having the most expansive contrast response function (Gouws et al., 2014) and well-known orientation-selective neurons (Hubel and Wiesel, 1968). Adding a high RF component that defined the RF-ripple stimuli had largely predictable effects on the tuning we measured from early visual areas. Because the addition of the ripple reduced the contrast and orientation variations across the stimuli and also changed the relationship of those variations with RF, the high RF tunings in early visual cortex shifted to a lower, although still remaining high, RF. The results fit well with the idea that contrast and orientation content of the stimuli are driving the responses in early visual cortex and that coding of global properties of shape is unlikely to be found in the neural responses within these regions (Wilkinson et al., 2000).

### Stimulus properties that drive responses in object-selective cortex

By contrast, tuning to low RFs, whether they occurred in the RF or the RF-ripple stimuli, while rarer overall, were found consistently in object-selective LO (Kourtzi and Kanwisher, 2000; Grill-Spector et al., 2001). The global properties of shape to which humans are exquisitely sensitive (Wilkinson et al., 1998) are therefore a feature of neural response in LO. LO exhibits visual field biases (Silson et al., 2016) but is not routinely identified with retinotopic mapping techniques. Indeed, in the present study, and in line with many others, LO was identified on the basis of a preference to objects over their scramble counterparts (Kourtzi and Kanwisher, 2000; Grill-Spector et al., 2001). The tunings to global shape properties are therefore consistent with the more abstract representations that have been associated with this region of the brain in previous research (Haushofer et al., 2008; Op de Beeck et al., 2008; Drucker and Aguirre, 2009). It is interesting, however, that the two retinotopically identified regions LO-1 and LO-2 (Larsson and Heeger, 2006) also exhibit tunings to low RF, which is consistent with our previous work that has shown a transition from early local processing of shape profile to processing of the complexity of shape at the same stage of the visual hierarchy (Vernon et al., 2016). The selectivity to global shape properties, which were largely maintained across our two stimulus sets, was also underscored by the multivariate patterns of response that were significantly correlated across the stimulus sets only in LO-1, LO-2, and LO.

### Is there a role for V4 in global shape processing?

V4 has been the focus of a body of research into properties that contribute to the processing of shape (for review, see Pasupathy et al., 2020). Work on nonhuman primates highlights the processing of curvature and more complex forms (Gallant et al., 1993, 1996). Recent work has led to the compelling idea that V4 is “shape emergent” (Hu et al., 2020). However, studies also highlight that the curvature selective domains in V4 intermingle with domains selective to orientation and chromatic stimulus properties (Roe et al., 2012; Hu et al., 2020; Tang et al., 2020). With that backdrop, it is clear that the stimuli we presented in the RF study had orientation variations that could easily have driven orientation-selective responses in V4 and masked selectivity to curvature and/or global shape. Consistent with this, the responses to low RFs emerged in V4 when much of the variation in orientation content of the stimuli was removed in the RF-ripple stimuli. The evidence we have gathered here therefore is consistent with V4 being a shape-emergent (Hu et al., 2020) region of the brain, albeit one that processes other stimulus properties as well. There is also good evidence in human that V4 plays an important role in shape processing (Wilkinson et al., 2000; Dumoulin and Hess, 2007). The stimuli used by Dumoulin and Hess (2007) are well suited to disentangle the contributions of orientation of stimulus elements and their arrangement into contours, and this could have contributed to them showing curvature processing in V4. They also reported curvature processing in a region that likely coincided with LO-1 and LO-2.

### Areas of the brain that represents global shape defined by RF

Previous work that was similar to ours has shown that RF is not represented in the regions of the ventral visual pathway that we have examined here (Salmela et al., 2016). Our study differed in ways that control for variations in low-level properties of RF stimuli that were present in the previous study (Salmela et al., 2016) and as a result likely explains why our investigation found processing and representations of RF in the regions that they did not. Here, we show that RF is represented in LO on the basis of differences in RF that approximate to how they are perceived: log ratios predicted the pattern of response better than differences in RF. This points to LO being a region that not only processes the global curvature found in RF patterns, but also represents that information in a way that has a plausible relationship with behavior, as also demonstrated using different shape stimuli (Haushofer et al., 2008; Op de Beeck et al., 2008; Drucker and Aguirre, 2009). LO-1 and LO-2 also showed representations that were related well to log ratios of the RF, although not as strongly as in LO. This indicates that representations of global shape emerge earlier than LO in the hierarchy of the ventral pathway, consistent with our previous work (Vernon et al., 2016).

In conclusion, our univariate results show clearly that two different response profiles were present in visual cortex. One showed a preference to high RF patterns, which we believe are driven by neural responses to local features, while the second showed preference to low RFs consistent with more global shape processing. The extent to which these response profiles were found in different regions of visual cortex varied markedly. Indeed, tuning to low RFs was localized to area LO, and this was unaffected by the addition of high RF contour modulations in the control rippled stimuli, suggesting a critical role in global shape processing. The multivariate analysis of the data also pointed to there being a global shape representation in lateral occipital regions that related well to a proxy of the perceptual differences between the stimuli, underscoring the role of lateral occipital regions in global shape processing.
